# Complete genome sequence of *Arthrobacter sp.* strain FB24

**DOI:** 10.4056/sigs.4438185

**Published:** 2013-09-30

**Authors:** Cindy H. Nakatsu, Ravi Barabote, Sue Thompson, David Bruce, Chris Detter, Thomas Brettin, Cliff Han, Federico Beasley, Weimin Chen, Allan Konopka, Gary Xie

**Affiliations:** 1 Department of Agronomy, Purdue University, West Lafayette, IN; 2 Los Alamos National Laboratories, Los Alamos, NM; 3 Pacific Northwest National Laboratory, Richland, WA

## Abstract

*Arthrobacter sp.* strain FB24 is a species in the genus *Arthrobacter* Conn and Dimmick 1947, in the family *Micrococcaceae* and class *Actinobacteria*. A number of *Arthrobacter* genome sequences have been completed because of their important role in soil, especially bioremediation. This isolate is of special interest because it is tolerant to multiple metals and it is extremely resistant to elevated concentrations of chromate. The genome consists of a 4,698,945 bp circular chromosome and three plasmids (96,488, 115,507, and 159,536 bp, a total of 5,070,478 bp), coding 4,536 proteins of which 1,257 are without known function. This genome was sequenced as part of the DOE Joint Genome Institute Program.

## Introduction

*Arthrobacter sp.* strain FB24 was isolated from a microcosm made from soil collected at an Indiana Department of Transport facility in Seymour, Indiana. This site was of particular interest because the soils were contaminated by mixed waste, both petroleum hydrocarbons and extreme metal (chromium and lead) levels [1]. Details of microcosm enrichment and isolation procedures used to obtain the *Arthrobacter* strain have been described previously [2]. This isolate was of particular interest because of its extreme resistance to chromate [3,4]. This work is a part of a larger study determining the compositional and functional diversity of bacterial communities in soils exposed to long-term contamination with metals [5-7].

## Classification and features

**Figure 1:**
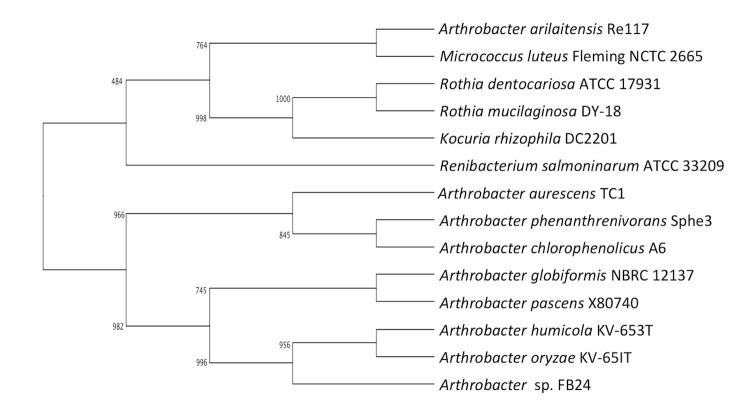
Phylogenetic tree of *Arthrobacter* strain FB24 relative to nearest neighboring *Arthrobacter* type strains and *Micrococcaceae* strains with finished genome sequences: *A. arilaitensis* re117 (FQ311476) [8], *A. aurescens* TC1 (NC_008709) [9], *A. chlorophenolicus* A6 (NC_011886), *A. phenanthrenivorans* Sphe3 (CP002379 [10], *Kocuria rhizophila* DC2201, *Microccus luteus* Fleming NCTC 2665, *Renibacterium salmoninarum* ATCC 33209, *Rothia dentocariosa* ATCC 17931, and *Rothia mucilaginous* DY-18. The sequences were aligned in ClustalX and a consensus tree was generated using a 1,000× repeated bootstrapping process [11,12].

**Figure 2:**
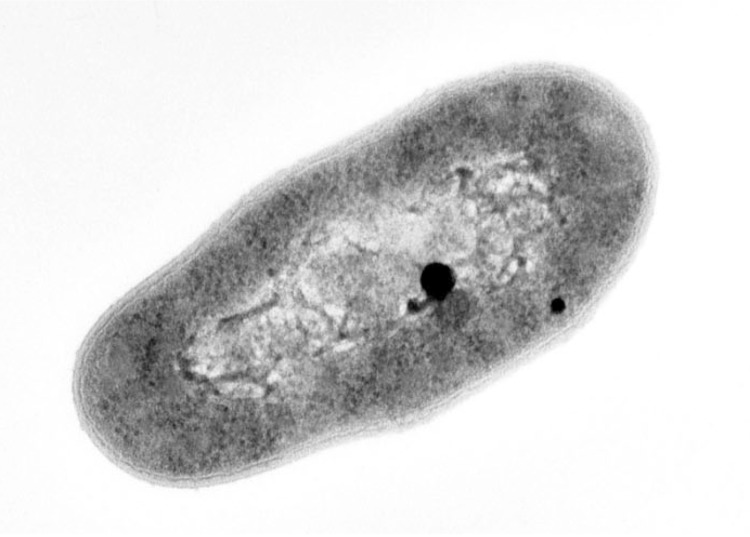
Transmission electron micrograph of *Arthrobacter sp.* strain FB24. Cells were grown in nutrient broth for 15 h (~early stationary phase), fixed in 3% glutaraldehyde in 0.1 M cacodylate buffer, then fixed in reduced osmium, followed by a series of ethanol dehydration steps. Cells are then embedded in Spurr resin, stained with uranyl acetate and Reynold’s lead citrate. Image was captured on Kodak SO-163 film at 33,000× magnification.

*Arthrobacter sp.* strain FB24 is a high G+C Gram-positive member of the *Micrococcaceae* (Figure 1, Table 1). The strain is a facultative, non-motile aerobe with characteristic morphology of rod-shaped cells (Figure 2) that become coccoid in stationary phase. Strain FB24 is able to use a number carbon sources for growth, including glucose, fructose, lactate, succinate, malate, xylose and aromatic hydrocarbons (hydroxybenzoates, phthalate). Additionally, this *Arthrobacter sp.* strain is resistant to multiple metals: arsenate, arsenite, chromate, cadmium, lead, nickel, and zinc.

**Table 1:** Classification and general features of *Arthrobacter* strain FB24

**MIGS ID**	**Property**	**Term**	**Evidence code**^a^
		Domain *Bacteria*	TAS [13]
		Phylum *Actinobacteria*	TAS [14]
		Class *Actinobacteria*	TAS [15]
	Current classification	Order *Actinomycetales*	TAS [15-18]
		Family *Micrococcaceae*	TAS [15-17,19]
		Genus *Arthrobacter*	TAS [17,20-23]
		Species *Arthrobacter sp.*	TAS [14]
		Type strain	TAS [15]
	Gram stain	Positive	IDA
	Cell shape	Polymorphic: Coccus to rod shape	IDA
	Motility	Non-motile	IDA
	Sporulation	Non-sporulating	IDA
	Temperature range	4-37°C	IDA
	Optimum temperature	30°C	IDA
	Carbon source	Yeast extract, glucose, fructose, lactate, succinate, malate, xylose, hydroxybenzoates, phthalate	
	Energy source	Yeast extract, glucose, fructose, lactate, succinate, malate, xylose, hydroxybenzoates, phthalate	
	Terminal electron receptor	Oxygen or nitrate	IDA
MIGS-6	Habitat	Soil	TAS [1]
	Isolation	Chromate and xylene enriched microcosm composed of anthropogenically disturbed soils	TAS [2]
MIGS-6.3	Salinity		
MIGS-22	Oxygen	Facultative aerobe	IDA
MIGS-15	Biotic relationship	Free-living	IDA
MIGS-14	Pathogenicity	Non-pathogenic	NAS
MIGS-4	Geographic location	Seymour, Indiana, USA	TAS [1,2]
MIGS-5	Sample collection time	June 27, 2001	IDA
MIGS-4.1	Latitude	38.9591667	NAS
MIGS-4.2	Longitude	-85.8902778	NAS
MIGS-4.3	Depth	40-90 cm	NAS
MIGS-4.4	Altitude	583 feet	NAS

^a^Evidence codes - IDA: Inferred from Direct Assay; TAS: Traceable Author Statement (i.e., a direct report exists in the literature); NAS: Non-traceable Author Statement (i.e., not directly observed for the living, isolated sample, but based on a generally accepted property for the species, or anecdotal evidence). These evidence codes are from the Gene Ontology project [24].

## Genome sequencing information

### Genome project history

*Arthrobacter sp.* strain FB24 was chosen for sequencing by DOE-JGI because of its extreme resistance to chromate. Table 2 presents the project information and its association with MIGS version 2.0 compliance [25].

**Table 2:** Project information

**MIGS ID**	**Property**	**Term**
MIGS-31	Finishing quality	Finished
MIGS-28	Libraries used	Small and medium random shotgun clones
MIGS-29	Sequencing platforms	Sanger
MIGS-31.2	Fold coverage	~15-fold
MIGS-30	Assemblers	Parallel PHRAP
MIGS-32	Gene calling method	Critica, Generation, Glimmer
	Genome Database release	March 1, 2007
	Genbank ID	12640
	Genbank Date of Release	October 24, 2006
	GOLD ID	Gc00445
	Project relevance	Bioremediation, biotechnological, environmental

### Growth conditions and DNA isolation

The FB24 culture used for DNA extraction was started from the glycerol stock (stored at -80 ºC) that was made from the original isolate. Cells were streaked onto a 0.1× nutrient agar plate, incubated at 30ºC, then a single colony was used to grow a culture in 0.25× nutrient broth (NB) (Difco, USA). Total genomic DNA was extracted from cells grown in liquid culture using the standard CTAB procedure [26].

### Genome sequencing and assembly

The random shotgun method was used in Sanger sequencing the genome of *Arthrobacter sp.* strain FB24 at the DOE-Joint Genome Institution (DOE-JGI). Medium (8 kb) and small (3 kb) insert random libraries were partially sequenced with average success rate of 88% and average high-quality read lengths of 614 nucleotides. Sequences were assembled with parallel phrap (High Performance Software, LLC). Possible mis-assemblies were corrected with Dupfinisher [27] or by analysis of transposon insertions in bridge clones. Gaps between contigs were closed by editing, custom primer walk or PCR amplification. The completed genome sequence of *Arthrobacter sp.* FB24 contains 89530 reads, achieving an average of 15-fold sequence coverage per base with an error rate less than 1 in 100,000. The sequences of *Arthrobacter sp.* FB24 can be accessed using the GenBank accession number NC_008541 for the chromosome and NC_008537, NC_008538, NC_008539 for three plasmids.

### Genome annotation

Automated gene prediction was performed by using the output of Critica [28], combined with the output of Generation and Glimmer [29]. The assignment of product descriptions was made by using search results of the following curated databases in this order: TIGRFam; PRIAM (*e*^–30^ cutoff); Pfam; Smart; COGs; Swissprot/TrEMBL (SPTR); and KEGG. If there was no significant similarity to any protein in another organism, it was described as “hypothetical protein.” “Conserved hypothetical protein” was used if at least one match was found to a hypothetical protein in another organism. EC numbering was based on searches in PRIAM at an *e*^–10^ cutoff; COG and KEGG functional classifications were based on homology searches in the respective databases. Additionally, the tRNAScanSE tool [30] was used to find tRNA genes, whereas ribosomal RNAs were found by using BLASTn vs. the 16S and 23S ribosomal RNA databases. Other “standard” structural RNAs (e.g., 5S rRNA, rnpB, tmRNA, SRP RNA) were found by using covariance models with the Infernal search tool [31]. The HMMTOP program was used to predict the number of transmembrane segments (TMSs) in each protein. Those predicted to have two or more TMSs (about 918 proteins) were used to interrogate the transporter database (TCDB). Peter Karp’s pathologic tool was used for pathway prediction [32]. This method largely relies on the keyword matching and other automatic methods to manually curate some of the pathways, such as aromatic compound degradation. Metabolic pathways were constructed using MetaCyc as a reference data set [33].

## Genome properties

**Figure 3:**
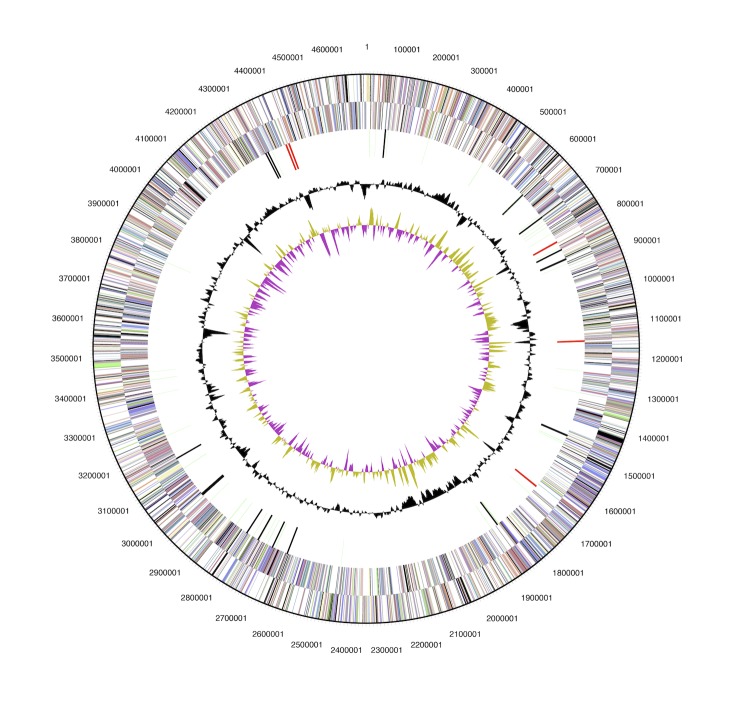
Circular map of FB24 chromosome, graphical depiction from outside to the center: genes on forward strand, genes on reverse strand (colored by COG categories), RNA genes (tRNAs green, rRNAs red, other RNAs black), GC content, GC skew. Chromosome is not to scale with plasmid maps.

**Figure 4:**
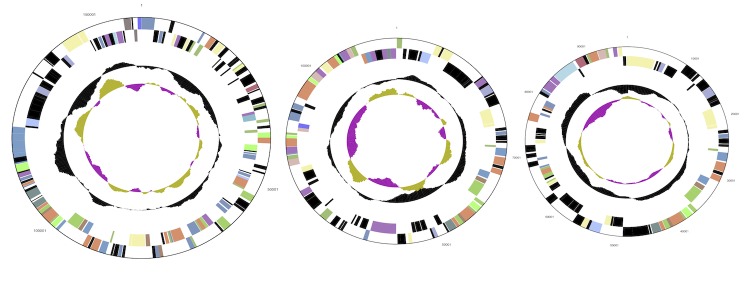
Circular map of three plasmids in FB24, graphical depiction from outside to the center: genes on forward strand, genes on reverse strand (colored by COG categories), RNA genes (tRNAs green, rRNAs red, other RNAs black), GC content, GC skew. Plasmid maps not to scale with each other or with chromosome map.

The 5,070,478- base pair genome of *Arthrobacter FB24* is composed of a single 4,698,945-base pair circular chromosome and three large circular plasmids (96,488, 115,507, and 159,536 bp) (Table 3) with GC content of 65.5, 64.7, 63.3 and 65.0%, respectively. Based on a summary of genomic features listed on the Integrated Microbial Genomes (IMG) [34] there are 4,536 protein coding sequences identified, of which 3,279 (70.94%, Table 4) have been assigned to a COG functional category (Table 5, Figure 3and Figure 4). There are 1,257 (27.19%) predicted genes without an associated function.

**Table 3:** Summary of genome

**Label**	**Size (bp)**	**Topology**	**INSDC identifier**	**RefSeq ID**
Chromosome 1	4,698,945	Circular	CP000454.1	NC_008541.1
Plasmid pFB104	96,488	Circular	CP000457.1	NC_008539.1
Plasmid pFB105	115,507	Circular	CP000456.1	NC_008538.1
Plasmid pFB136	159,536	Circular	CP000455.1	NC_008537.1

**Table 4:** Nucleotide content and gene count levels of the genome

**Attribute**	Value	% of total^a^
Genome size (bp)	5,070,478	100.0
DNA coding region (bp)	4,552,065	89.78
DNA G+C content (bp)	3,315,507	65.39
Total genes^b^	4,622	100.00
RNA genes	86	1.86
Protein-coding genes with function prediction	3,279	70.94
Protein coding genes without function prediction	1,257	27.19
Genes in paralog clusters	965	20.88
Genes assigned to COGs	3,361	72.72
Genes with signal peptides	1,098	23.76
Genes with transmembrane helices	1,168	25.27
Paralogous groups	373	100.00

a) The total is based on either the size of the genome in base pairs or on the total number of protein coding genes in the annotated genome.

b) Also includes 54 pseudogenes and 5 other genes

**Table 5:** Number of genes associated with general COG functional categories

**Code**	**Value**	**%age^a^**	**Description**
J	162	4.27	Translation, ribosomal structure and biogenesis
A	1	0.03	RNA processing and modification
K	363	9.57	Transcription
L	164	4.32	Replication, recombination and repair
B	1	0.03	Chromatin structure and dynamics
D	32	0.84	Cell cycle control, cell division, chromosome partitioning
Y	-	-	Nuclear structure
V	49	1.29	Defense mechanisms
T	162	4.27	Signal transduction mechanisms
M	171	4.51	Cell wall/membrane/envelope biogenesis
N	3	0.08	Cell motility
Z	1	0.03	Cytoskeleton
W	0	0.0	Extracellular structures
U	48	1.27	Intracellular trafficking, secretion, and vesicular transport
O	124	3.27	Posttranslational modification, protein turnover, chaperones
C	239	6.3	Energy production and conversion
G	436	11.49	Carbohydrate transport and metabolism
E	364	9.6	Amino acid transport and metabolism
H	98	2.58	Nucleotide transport and metabolism
I	155	4.09	Lipid transport and metabolism
P	207	5.46	Inorganic ion transport and metabolism
Q	112	2.95	Secondary metabolites biosynthesis, transport and catabolism
			
R	458	12.07	General function prediction only
S	286	7.54	Function unknown
-	1,261	27.28	Not in COG

a) The total is based on the total number of protein coding genes in the annotated genome.

## Genome comparisons

**Figure 5:**
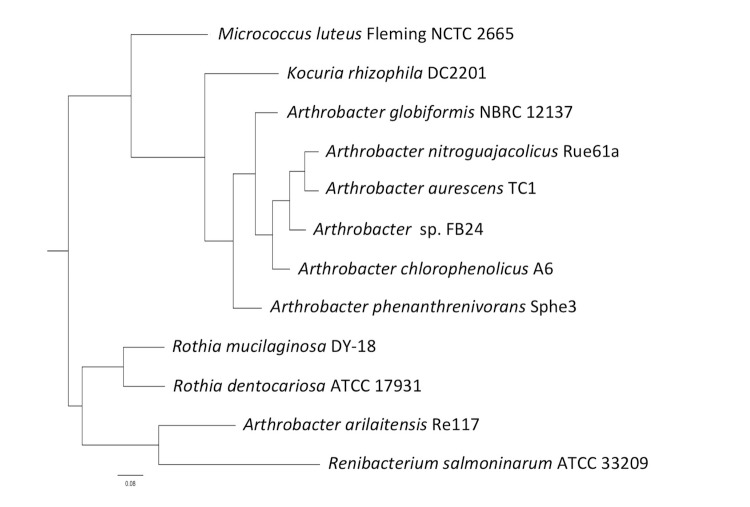
Hierarchical tree based on similarity of COG groups between genomes. Included are genomes of bacteria in the family *Micrococcaceae* with finished genome sequences.

A comparative analysis of genome sizes and protein coding genes in *Arthrobacter sp.* FB24 and other *Arthrobacter* species with finished sequences (Table 6) was made from data listed on the IMG website [34]. Included in the comparison is *A. arilaitensis* re117 (Gc01419, FQ311476) [8], *A. aurescens* TC1 (Gc00480, NC_008709) [9], *A. chlorophenolicus* A6 (Gc00930, NC_011886), *A. nitroguajacolicus* Rue61a (Gc0006272, CP003203), and *A. phenanthrenivorans* Sphe3 (Gc01621, CP002379) [10]. In addition, the draft genome of *A. globiformis* NBRC 12137 was included because its phylogenetic relatedness to FB24 based on the 16S rRNA gene sequence. Similarity between functional protein groups (based on COG, clusters of orthologous groups) in the genomes of these strains were made and visualized using hierarchical clustering (Figure 5) with tools available on the Joint Genome Institute (JGI) Integrated Microbial Genomes (IMG) site. Also included in the tree were closely related species in the family *Micrococcaceae* with finished genomes *Kocuria rhizophila* DC2201 (Gc00769), *Microccus luteus* Fleming NCTC 2665 (Gc01033), *Renibacterium salmoninarum* ATCC 33209 (Gc00698), *Rothia dentocariosa* ATCC 17931 (Gc01662), and *Rothia mucilaginosa* DY-18 (Gc01162). Detailed information about the genome properties and genome annotation of these strains can be obtained from the JGI-IMG website at the JGI website [35].

**Table 6:** Comparison of genomes of the genus *Arthrobacter* with finished genome sequences

Genome Name	Genome size (bp)	Gene count	Protein coding	Protein with function	Without function	Plasmid number	rRNA operons
*Arthrobacter arilaitensis* re117, CIP108037	3,918,192	3,518	3,436	2,390	1,046	2	6
* *							
*Arthrobacter aurescens* TC1	5,226,648	4,793	4,699	3,419	1,280	2	6
* *							
*Arthrobacter chlorophenolicus* A6	4,980,870	4,744	4,641	3,125	1,516	2	5
* *							
*Arthrobacter nitroguajacolicus* Rue61a	5,081,038	4,655	4,584	3,800	784	2	6
* *							
*Arthrobacter phenanthrenivorans* Sphe3	4,535,320	4,273	4,209	3,101	1,108	2	4
* *							
*Arthrobacter sp.* FB24	5,070,478	4,622	4,536	3,279	1,257	3	5
* *							
*Arthrobacter globiformis* NBRC 12137*	4,954,410	4,582	4,529	2,784	1,745	?	1

*Sequence not fully assembled
